# Optimizing resource allocation: Cost-effectiveness of specified D-dimer cut-offs in cancer patients with suspected venous thromboembolism

**DOI:** 10.1007/s11239-024-03000-2

**Published:** 2024-05-18

**Authors:** Teodora Biciusca, Leon D. Gruenewald, Simon S. Martin, Jennifer Gotta, Scherwin Mahmoudi, Katrin Eichler, Christian Booz, Christian Salbach, Matthias Müller-Hennessen, Moritz Biener, Mustafa Yildirim, Barbara Milles, Christof M. Sommer, Thomas J. Vogl, Evangelos Giannitsis, Vitali Koch

**Affiliations:** 1 Department of Diagnostic and Interventional Radiology, Goethe University Frankfurt, University Hospital, Frankfurt Am Main, Germany; 2 Department of Cardiology, Angiology, and Pulmonology, University of Heidelberg, University Hospital, Heidelberg, Germany; 3 Department of Diagnostic and Interventional Radiology, University of Heidelberg, University Hospital, Heidelberg, Germany

**Keywords:** Deep vein thrombosis, Pulmonary embolism, Venous thromboembolism, D-dimer, Cost-effectiveness analysis, Cancer

## Abstract

An accurate diagnosis of venous thromboembolism (VTE) is crucial, given the potential for high mortality in undetected cases. Strategic D-dimer testing may aid in identifying low-risk patients, preventing overdiagnosis and reducing imaging costs. We conducted a retrospective, comparative analysis to assess the potential cost savings that could be achieved by adopting different approaches to determine the most effective D-dimer cut-off value in cancer patients with suspected VTE, compared to the commonly used rule-out cut-off level of 0.5 mg/L. The study included 526 patients (median age 65, IQR 55–75) with a confirmed cancer diagnosis who underwent D-dimer testing. Among these patients, the VTE prevalence was 29% (n = 152). Each diagnostic strategy's sensitivity, specificity, negative likelihood ratio (NLR), as well as positive likelihood ratio (PLR), and the proportion of patients exhibiting a negative D-dimer test result, were calculated. The diagnostic strategy that demonstrated the best balance between specificity, sensitivity, NLR, and PLR, utilized an inverse age-specific cut-off level for D-dimer [0.5 + (66-age) × 0.01 mg/L]. This method yielded a PLR of 2.9 at a very low NLR for the exclusion of VTE. We observed a significant cost reduction of 4.6% and 1.0% for PE and DVT, respectively. The utilization of an age-adjusted cut-off [patient’s age × 0.01 mg/L] resulted in the highest cost savings, reaching 8.1% for PE and 3.4% for DVT. Using specified D-dimer cut-offs in the diagnosis of VTE could improve economics, considering the limited occurrence of confirmed cases among patients with suspected VTE.

**Graphical Abstract:**

In the context of accurate diagnosis of VTE, strategic D-dimer testing helps identify low-risk patients, preventing overdiagnosis and reducing imaging costs. In our retrospective study, the diagnostic strategy that demonstrated the best balance between specificity, sensitivity, and best PLR, utilized an inverse age-specific cut-off level for D-dimer. We observed a significant cost reduction of 4.6% for PE and 1% for DVT. *Abbreviations: CTPA, computed tomography pulmonary angiography; CUS, compression ultrasound; DVT, deep vein thrombosis; PE, pulmonary embolism; VTE, venous thromboembolism.*

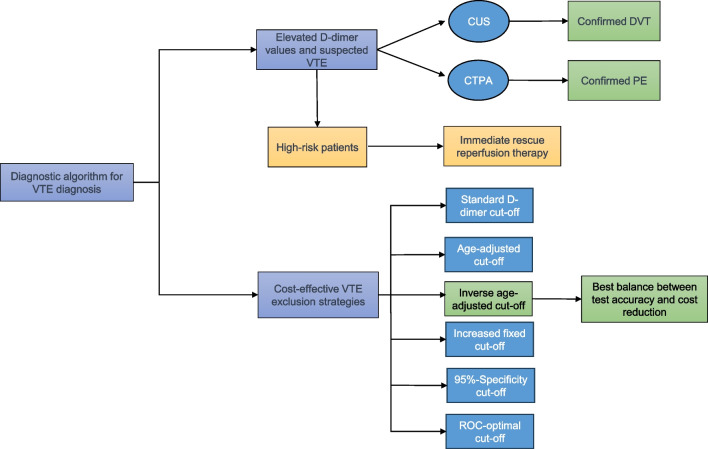

## Introduction

Deep vein thrombosis (DVT) and pulmonary embolism (PE) represent the primary manifestations of venous thromboembolism (VTE), a complex and significant medical entity that stands as the third most prevalent cause of cardiovascular mortality, surpassed only by myocardial infarction and stroke in its impact on public health [1–4].

The occurrence of VTE is a complex event influenced by various factors, including both patient-specific factors (dispositional factors) and external risk factors (exposure) [5–7]. In cancer patients, these factors are frequently aggravated by the pro-coagulant impact of cancer therapies and tumor biology. Consequently, individuals with cancer face a roughly eight-fold higher likelihood of experiencing VTE compared to those who do not have cancer [1]. This escalated risk of VTE in cancer patients is linked to unfavorable implications on their survival and is regarded as a noteworthy contributor to mortality [8–15].

Precise diagnosis of VTE holds paramount importance, given the considerable risks of morbidity and mortality that may arise from undetected cases. Furthermore, it is important to consider the potential adverse effects, logistical challenges, and resource demands associated with diagnostic procedures and anticoagulant therapy for VTE [16, 17]. Despite technological progress, radiological procedures continue to entail certain risks, notably related to radiation and contrast agent exposure, which raises concerns about their overuse. In response, diagnostic algorithms have been developed to establish standardized protocols and reduce the incidence of unnecessary and potentially distressing procedures. These algorithms integrate various clinical assessment tools, including the widely used Wells score, alongside D-dimer testing and diagnostic imaging methods like computed tomography pulmonary angiography (CTPA) or compression ultrasound (CUS) [1]. The implementation of D-dimer testing serves the purpose of effectively identifying patients with low clinical probability, thereby reducing the need for additional imaging. Remarkably, normal D-dimer results offer a high degree of certainty in ruling out DVT [18]. However, it is crucial to recognize that an elevation in D-dimer does not automatically signify the presence of thromboembolism. D-dimers are increased in various conditions, such as cancer or even advanced age [1].

In recent years, systematic economic evaluations have gained popularity, recognizing the pivotal role of cost-effectiveness analysis in assessing the affordability and resource implications of recommended strategies [16, 19–24]. Numerous cost-effectiveness analyses have been conducted to compare various diagnostic approaches involving D-dimer and the use of CTPA with alternative methods. Overall, these investigations have suggested that pairing D-dimer testing with another moderately expensive strategy may not only enhance diagnostic performance but also prove to be cost-effective and sometimes even cost-saving [16, 25–28].

In our study, we assessed potential cost savings linked to six different D-dimer testing strategies among cancer patients, both with and without VTE. Our objective was to identify the most cost-effective approach for accurately diagnosing VTE. The insights obtained from our study, along with existing guideline recommendations, have the potential to facilitate the adoption of timely and cost-effective diagnostic testing strategies for individuals suspected of having VTE.

## Methods

### Study population

In this study, we conducted a comparative cost-effectiveness analysis of six different diagnostic strategies for the exclusion of VTE by D-dimer testing, utilizing data from a study conducted by Koch et al. in 2022 [11]. Our study population consisted of 526 cancer patients with suspected VTE who were recruited in the Chest Pain Unit of the University of Heidelberg (Heidelberg, Baden-Wuerttemberg, Germany). It is important to note that the cancer diagnosis preceded the occurrence of thromboembolism and the subsequent referral to the emergency department. The comparative group of cancer patients without the final adjudicated diagnosis of VTE encompassed a diverse array of conditions, including vascular diseases such as peripheral arterial disease and cardiac disease (e.g., hypertensive crisis, acute myocardial infarction), respiratory diseases (e.g., pneumonia, chronic obstructive pulmonary disease, bronchial asthma), gastrointestinal diseases (e.g., gastritis, gastroesophageal reflux), and orthopedic conditions (e.g., joint pain, arthritis, trauma) [11].

### Diagnostic procedure

According to current guidelines, the diagnostic strategy for the exclusion of VTE involved a series of sequential steps within the emergency department [1]. This approach typically included a physical examination, an electrocardiogram (ECG), CUS or CTPA, and laboratory tests such as a complete blood count, creatinine assessment, D-dimer testing, and prothrombin time evaluation [11].

Upon admission, comprehensive data were gathered, encompassing patient characteristics, medical history, physical examination findings, diagnostic test results, and treatment details. In this analysis, the patients were categorized into distinct groups based on their pretest probability (PTP) of having PE or DVT, using the three-level Wells scores (< 2 points: low probability; 2–6 points: medium probability; ≥ 7 points: high probability). D-dimer testing was performed at the discretion of the attending physician immediately after the patient’s admission to the emergency department. If the D-dimer test yielded a negative result, it was considered evidence of excluding VTE, and no imaging or anticoagulant treatment had been initiated [11].

**Fig. 1:**
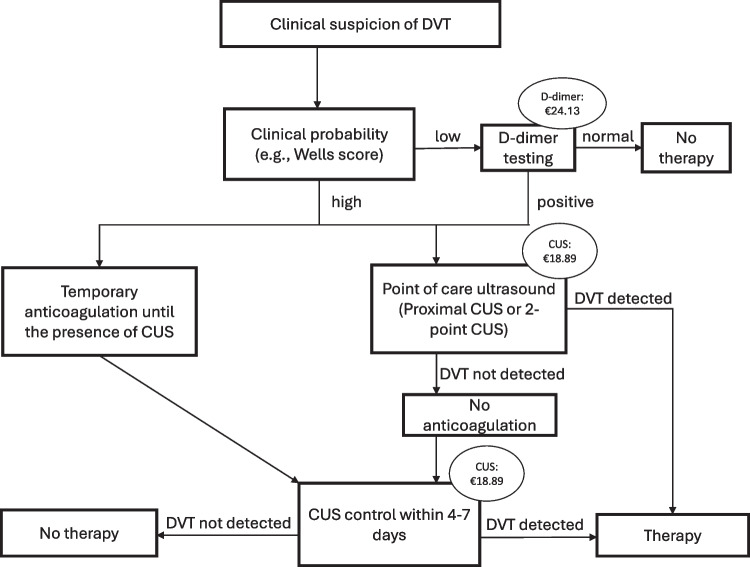
Diagnostic algorithm for suspected DVT (first event) using compression ultrasonography, adapted according to current guidelines [1]. Pricings for CUS and D-dimer testing were taken from the German scale of fees for physicians (MFS) ^51^. *Abbreviations: CUS, compression ultrasound; DVT, deep vein thrombosis; MFS, Medical fee schedule*

**Fig. 2:**
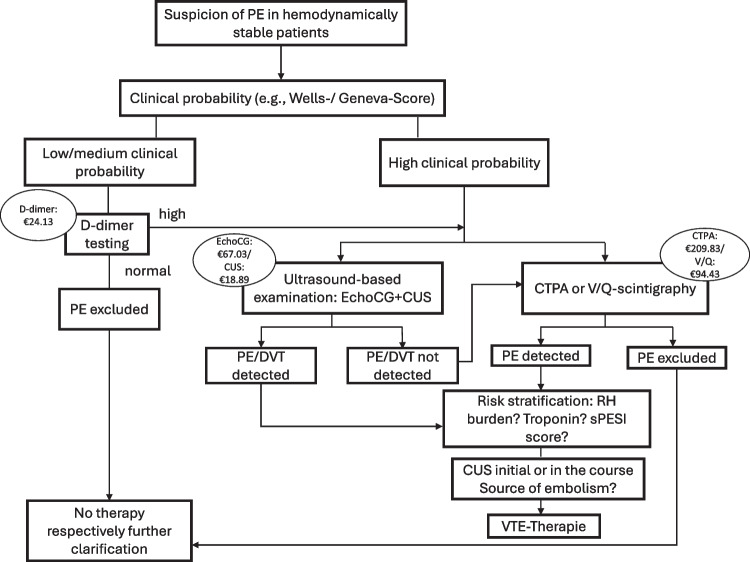
Diagnostic algorithm for suspected PE in hemodynamically stable patients, adapted according to current guidelines [1]. All prices were taken from the German scale of fees for physicians (MFS) ^51^. *Abbreviations: CUS, compression ultrasound; CTPA, computed tomography pulmonary angiography; DVT, deep vein thrombosis; EchoCG, echocardiogram; MFS, Medical fee schedule; PE, pulmonary embolism; RH, right heart; sPESI, simplified Pulmonary Embolism Severity Index; VTE, venous thromboembolism; V/Q-scintigraphy, ventilation-perfusion scintigraphy*

Following the institutional protocol and latest guidelines, patients with elevated D-dimer values and suspected VTE were further evaluated using either CUS or CTPA, except for high-risk patients with hemodynamic compromise, who received immediate rescue reperfusion therapy. Patients were deemed positive for VTE if they exhibited confirmed cases of PE or proximal DVT, as identified through diagnostic imaging tests. Figures 1 and 2 illustrate the diagnostic algorithm used for diagnosing VTE in our study.

To determine the most cost-effective method for VTE exclusion, we compared a conventional diagnostic strategy using the commonly recommended D-dimer rule-out cut-off 0.5 mg/L [1] (Method 1) together with five other methods based on different approaches to establish an optimal cut-off level for VTE exclusion according to current literature:

Method 2 (Age-adjusted cut-off [patient’s age × 0.01 mg/L]) [1]: The determination of the age-adjusted cut-off level was performed by multiplying the patient’s age by 10 in individuals who were over 50 years old. In this age-adjusted strategy, patients up to 50 years of age were considered negative for PE if their D-dimer levels were below 0.5 mg/L, while patients over 50 years of age were considered negative if their D-dimer levels were below 10 times their age.

Method 3 (Inverse age-adjusted cut-off [0.5 + (66-age) × 0.01 mg/L]): The establishment of the cut-off level in patients below 66 years was based on an age-adjusted criterion that operated inversely [11, 29, 30].

Method 4 (Increased fixed cut-off [1 mg/L]): The selection of the cut-off level was based on a higher fixed threshold of 1 mg/L [11, 30].

Method 5 (95%-Specificity cut-off [4.9 mg/L]): The selection of this cut-off level was based on a specificity of 95% that yielded a diagnostic threshold of 4.9 mg/L [11].

Method 6 (Receiver operating characteristic [ROC]-optimal cut-off [9.9 mg/L]): Prespecified ROC-optimized cut-off at 9.9 mg/L balancing sensitivities and specificities to establish an optimal detection threshold [11].

### D-dimer assessment

D-dimers were assessed in the central laboratory using the Innovance D-dimer assay, which had a diagnostic threshold of 0.5 mg/L (Siemens Healthineers AG, Forchheim, Germany). This assay utilizes a particle-enhanced, immune-turbidimetric method to quantitatively measure D-dimers in human plasma on dedicated coagulation analyzers (CS-5100; Siemens Healthineers AG) [11].

### Economic evaluation

The analysis was conducted on the assumption that D-dimer was ordered for all patients with non-high PTP, and imaging tests were performed only when the test result exceeded the specified cut-off level of the respective diagnostic strategy. All monetary values utilized in the calculations were reported in € for Germany and in $ for the United States of America. In Germany, these expenses are invoiced using the German scale of fees for physicians (MFS, Medical fee schedule), which establishes standard fees for specific services. Accordingly, basic costs for CUS were €18.89, for D-dimer testing €24.13, and for CTPA €209.83 [31, 32]. Regarding the imaging costs in the US based on the published literature, which reflects the average expenses for patients with statutory health insurance, the prices were as follows: $184 for CUS, $14 for D-dimer testing, and $648 for CTPA [25, 33, 34].

To estimate the cost savings over one year, we adopted the presumption of a patient cohort consisting of 5475 individuals with cancer and suspected VTE (1825 with PE and 3650 with DVT), considering the data of our department (estimated average of 5 patients/day with PE and 10 patients/day with DVT).

### Statistical analysis

The statistical analysis was conducted using MedCalc software (MedCalc Software Ltd., Version 22.016, Ostend, Belgium) [11]. The normality of data distribution was evaluated using the Kolmogorov–Smirnov test. Based on this, continuous variables were presented as mean ± standard deviation (SD) or as median with 25th/75th percentiles (interquartile range, IQR). Categorical variables were reported as numbers with corresponding percentages. To compare the D-dimer test results within the same group of patients, we used the Wilcoxon signed-rank test for paired samples and evaluated the correlation between the D-dimer test results and other variables, such as patient characteristics, risk factors, or clinical outcomes using Spearman’s coefficient of rank correlation. P values < 0.05 were considered statistically significant. Each diagnostic strategy’s sensitivity, specificity, negative predictive value (NPV), positive predictive value (PPV), negative likelihood ratio (NLR), positive likelihood ratio (PLR), and the proportion of patients exhibiting a negative D-dimer test result were calculated and compared to those of the recommended cut-off level of 0.5 mg/L for ruling out VTE. Areas under the ROC curve (AUCs) were calculated using the methodology of DeLong. Additionally, ROC-optimized cut-offs were determined to strike a balance between sensitivities and specificities for identifying the optimal detection threshold for VTE.

## Results

### Study population

According to previously published data, D-dimer concentrations were measured in a cohort of 526 cancer patients, with a median age of 65 (range, 29–92; IQR, 55–75) [11]. Among these patients, 83 (16%) had PE, and 69 (13%) had DVT, resulting in a VTE prevalence of 29% (n = 152). Within the PE cases, 19% (16 out of 83) were classified as massive PE and were treated directly through hemodynamic stabilization, initiation of anticoagulation, and systemic thrombolysis. The occurrence of a VTE event was absent in 71% of the patients and these patients represented our control group [11].

The majority of patients with PE and DVT had T4 cancer, accounting for 55% and 39%, respectively. In contrast, among patients without a VTE event, the most common cancer stage was T2, representing 31% of cases.

Among the cancer patients included in this study, 37 individuals (7%) required intensive care treatment after being admitted to the emergency department. Table 1 provides a summary of patient characteristics.

**Table 1:** Demographics and outcome data of study patient population, adapted according to Koch et al. [11]

Variables - n (%) or median (IQR)	Overall (n = 526; 100%)	PE (n = 83; 16%)	DVT (n = 69; 13%)	w/o VTE (n = 374; 71%)	p-value
Demographics					
Age, years	65 (55–75)	65 (58–76)	63 (51–74)	65 (55–75)	0.1938
Male sex	297 (56%)	52 (63%)	30 (44%)	197 (53%)	
Female sex	229 (44%)	31 (37%)	39 (56%)	177 (47%)	
Clinical prediction rule					
Wells score (Original version)	2 (2–4)	6 (4–7)	4 (2–7)	2 (1–3)	< 0.0001
TNM classification					
T stage					
T1	122 (23%)	6 (7%)	9 (13%)	107 (29%)	
T2	141 (27%)	8 (10%)	19 (28%)	114 (31%)	
T3	124 (24%)	23 (28%)	14 (20%)	87 (23%)	
T4	139 (26%)	46 (55%)	27 (39%)	66 (18%)	
N stage					
N0	97 (18%)	15 (18%)	11 (16%)	71 (19%)	
N1	239 (45%)	35 (42%)	21 (30%)	183 (49%)	
N2	190 (36%)	33 (40%)	37 (54%)	120 (32%)	
M stage					
M0	400 (76%)	38 (46%)	44 (64%)	318 (85%)	
M1	126 (24%)	45 (54%)	25 (36%)	56 (15%)	
Type of care					
Inpatient (w/o intensive care)	124 (24%)	61 (74%)	24 (35%)	39 (10%)	
Intensive care	37 (7%)	8 (10%)	2 (3%)	27 (7%)	

*DVT* deep vein thrombosis; *IQR* interquartile range; *PE* pulmonary embolism; *VTE* venous thromboembolism; *w/o* without

### Characteristics of D-dimer testing

The D-dimer results in the study population are presented in Table 2. The median D-dimer level exhibited a significant difference (p < 0.0001) between the 152 patients with VTE and the 374 patients without VTE. Among the patients with VTE, the median D-dimer level was 7.4 mg/L (IQR, 3.7–11.4), while among those without VTE, it was 0.4 mg/L (IQR, 0.2–0.9). Of the 152 patients with VTE, all had D-dimer levels exceeding the conventional fixed cut-off level of 0.5 mg/L [11].

**Table 2:** D-dimer results expressed as median values with IQR, in cancer patients with confirmed VTE (n = 152) or excluded VTE (n = 374), divided into different age groups. Table adapted according to Koch et al. [11]

	Patients with VTE (n = 152)			Patients without VTE (n = 374)	
Variables—median (IQR)	D-dimer (mg/L	D-dimer below 0.5 mg/L (n, %)	D-dimer below 1.0 mg/L (n, %)	D-dimer (mg/L)	D-dimer below 0.5 mg/L (n, %)
Overall					
	7.4 (3.7–11.4) (n = 152)	0/152 (0%)	6/152 (4%)	0.4 (0.2–0.9) (n = 374)	219/374 (59%)
Stratified by age					
< 25	4.1 (n = 1)	-	0/1 (0%)	-	-
25–35	4.5 (3.9–6.1) (n = 5)	-	0/5 (0%)	0.4 (n = 1)	1/1 (100%)
36–45	6.2 (3.1–10.5) (n = 10)	-	1/10 (10%)	0.3 (0.2–0.7) (n = 28)	19/28 (68%)
46–55	4.6 (2.3–9.0) (n = 25)	-	0/25 (0%)	0.5 (0.2–0.9) (n = 65)	37/65 (57%)
56–65	7.8 (4.7–14.3) (n = 35)	-	2/35 (6%)	0.3 (0.2–0.7) (n = 95)	59/95 (62%)
66–75	5.8 (3.6–10.1) (n = 29)	-	1/29 (3%)	0.3 (0.2–1.0) (n = 93)	54/93 (58%)
76–85	7.9 (4.6–11.5) (n = 41)	-	2/41 (5%)	0.4 (0.2–1.3) (n = 76)	41/76 (54%)
> 86	10.3 (6.9–13.1) (n = 6)	-	0/6 (0%)	0.5 (0.3–0.7) (n = 16)	8/16 (50%)

*IQR, interquartile range; VTE, venous thromboembolism*

The D-dimer level showed a significant correlation with age (r = 0.166, p = 0.0412). Consequently, both the median D-dimer level and the proportion of patients with D-dimer results above 0.5 mg/L increased with age in different age groups (as shown in Table 2), leading to an age-related decline in test specificity, particularly among individuals over 70 years old [11].

Regarding the specific type of cancer, individuals diagnosed with hematologic cancer displayed the highest levels of D-dimer (3.7 mg/L, IQR 0.5–7.7), whereas those with cancer of uncertain primary origin exhibited the lowest D-dimer concentrations (2.0 mg/L, IQR 0.8–3.7; p = 0.2246) [11].

### D-dimer testing strategies

The standard method (Method 1), the age-adjusted method (Method 2), and the inverse age-adjusted method (Method 3) all exhibited the highest sensitivity values, achieving ≥ 99%. These methods also demonstrated superior NPV, with all three achieving ≥ 99% (as shown in Table 3).

**Table 3:** Diagnostic performance of the Innovance D-dimer assay in 526 cancer patients with suspected VTE using different diagnostic D-dimer thresholds

D-dimer threshold	AUC-associated cut-off (mg/L)	Sensitivity (%)	Specificity (%)	AUC	AUC (95% CI)	PPV (%)	NPV (%)	PLR	NLR	p-value (AUC)	False negatives (n)
Method 1 Rule-out cut-off [0.5 mg/L]											
Overall VTE	0.5	100	65	0.942	0.92 – 0.96	54	100	2.8	0	< 0.0001	0
PE	0.5	100	65	0.950	0.93 – 0.97	39	100	2.8	0	< 0.0001	0
DVT	0.5	100	70	0.932	0.90 – 0.95	38	100	3.3	0	< 0.0001	0
Method 2											
Age-adjusted cut-off [patient's age × 0.01 mg/L]											
Overall VTE	0.7	100	45	0.942	0.92 – 0.96	42	100	1.8	0	< 0.0001	0
PE	0.8	100	70	0.950	0.93 – 0.97	43	100	3.3	0	< 0.0001	0
DVT	0.9	100	72	0.932	0.90 – 0.95	39	100	3.6	0	< 0.0001	0
Method 3											
Inverse age-adjusted cut-off [0.5 + (66-age) × 0.01 mg/L]											
Overall VTE	0.6	99	66	0.942	0.92 – 0.96	55	100	2.9	0	< 0.0001	0
PE	0.5	99	65	0.950	0.93 – 0.97	39	100	2.8	0	< 0.0001	0
DVT	0.6	100	70	0.932	0.90 – 0.95	38	99	3.3	0	< 0.0001	0
Method 4											
Increased fixed cut-off [1 mg/L]											
Overall VTE	1.0	96	77	0.943	0.92 – 0.96	63	98	4.2	0.1	< 0.0001	6
PE	1.0	95	77	0.952	0.93 – 0.97	48	99	4.1	0.1	< 0.0001	4
DVT	1.0	97	78	0.933	0.91 – 0.96	44	99	4.4	0.1	< 0.0001	2
Method 5											
95%-Specificity cut-off [4.9 mg/L]											
Overall VTE	4.9	64	95	0.942	0.92 – 0.96	83	87	12.8	0.4	< 0.0001	55
PE	5.1	70	95	0.950	0.93 – 0.97	75	93	14.0	0.3	< 0.0001	25
DVT	4.7	55	95	0.932	0.90 – 0.95	66	92	11.0	0.5	< 0.0001	30
Method 6											
ROC-optimal cut-off [9.9 mg/L]											
Overall VTE	9.9	30	100	0.942	0.92 – 0.96	96	78	-	0.7	< 0.0001	96
PE	9.9	36	100	0.950	0.93 – 0.97	94	88	-	0.6	< 0.0001	35
DVT	9.6	23	99	0.932	0.90 – 0.95	89	88	23.0	0.8	< 0.0001	52

*AUC, area under the curve; DVT, deep vein thrombosis; NLR, negative likelihood ratio; NPV, negative predictive value; PE, pulmonary embolism; PLR positive likelihood ration; PPV, positive predictive value; VTE, venous thromboembolism*

Except for the 95%-specificity cut-off (Method 5) and ROC-optimal cut-off (Method 6), almost all other diagnostic strategies showed comparably high sensitivities and NPVs when compared with the standard method, meeting the CLSI (Clinical and Laboratory Standards Institute) requirements for D-dimer assays used in VTE diagnosis: an NPV of at least 98% and a sensitivity of at least 97% [30].

The diagnostic strategy that demonstrated the best values for specificity, sensitivity, NLR, and PLR, utilized an inverse age-specific cut-off level for D-dimer (Method 3). This method demonstrated an NPV of 100% and specificity of 66% with a PLR of 2.9, comparable to those of the standard method (NPV of 100%, specificity of 65%, and PLR of 2.8). Furthermore, it demonstrated a remarkable lack of false positives with an NLR of virtually zero (0.01).

Method 6 yielded the greatest count of false negatives, encompassing a total of 96 cases (35 PE and 52 DVT). Conversely, method 5 produced a cumulative count of 55 false negatives, consisting of 25 for PE and 30 for DVT. These findings indicate that both methods are not suitable for accurately excluding VTE. In contrast, Method 4 exhibited only 6 false negatives, while the other methods did not produce any false negatives.

### Cost-effectiveness calculation

Method 6 has been excluded from cost-effectiveness analysis considering its high number of false negatives.

In terms of expenses, Table 4 illustrates that the most substantial cost savings were achieved through the adoption of Method 5. Method 3 proved to be the safest approach to exclude VTE in our study, with a PLR of 2.9 and an NLR of 0.01. This method resulted in savings of 24 CTPA and 5 CUS procedures. Although the savings were not as substantial as with method 5, the inverse age-adjusted cut-off method showed the best balance between specificity, sensitivity, and NPV. As a result, a total of €5131 could potentially be saved, with €5036 attributable to CTPA and €95 to CUS (4.6% for PE and 1% for DVT). The age-adjusted cut-off method (Method 2) resulted in the highest cost savings, totaling €9363, with €9023 allocated to CTPA and €340 to CUS (8.1% for PE and 3.4% for DVT). If we assume an annual case volume of 5475 patients with suspected VTE, the inverse age-adjusted method could lead to yearly savings of €53,400, while the age-adjusted method could result in savings of €97,454. If expenses for diagnostic procedures would be calculated with data from the United States, even more cost savings could be expected. The inverse age-adjusted method would result in savings of $15,552 for CTPA and $920 for CUS, for a total of $16,472. The age-adjusted method, on the other hand, would save $27,864 for CTPA and $3312 for CUS, for a total of $31,176. Consequently, with an annual case volume of 5475 patients, the inverse age-adjusted method could lead to savings of $171,453, whereas the age-adjusted method could achieve savings of $324,503.

**Table 4:** Economic analysis of different diagnostic strategies based on specified D-dimer thresholds

Specified cut-off classifier	Overall VTE (CTPA & CUS) (n)	Suspected PE (CTPA) (n)	Suspected DVT (CUS) (n)
Method 2			
Age-adjusted cut-off [patient's age × 0.01 mg/L]			
Number of saved examinations (vs. rule-out cut-off)	61	43	18
Method 3			
Inverse age-adjusted cut-off [0.5 + (66-age) × 0.01 mg/L]			
Number of saved examinations (vs. rule-out cut-off)	29	24	5
Method 4 Increased fixed cut-off [1 mg/L]			
Number of saved examinations (vs. rule-out cut-off)	67	49	18
Method 5			
95%-Specificity cut-off [4.9 mg/L]			
Number of saved examinations (vs. rule-out cut-off)	132	77	55

*CTPA computed tomography pulmonary angiography; CUS, compression ultrasound; DVT deep vein thrombosis; PE pulmonary embolism; VTE venous thromboembolism*

## Discussion

Due to the demographic shift towards an aging population, the frequency of suspecting PE involvement has increased over the last decade. However, confirmation of this suspicion is only found in a fraction of cases, with approximately 30% of cancer patients included in our study [35]. Achieving diagnostic certainty is crucial in hemodynamically stable patients to avoid both false-positive results for PE (sensitivity) and unnecessary examinations for patients without PE (specificity). The first step involves assessing clinical likelihood using established scores or empirical methods. Simplified versions of commonly used scores, such as the Wells score and revised Geneva score, are frequently employed in clinical practice [36, 37].

The risk of VTE events is approximately eight times higher in patients with cancer compared to individuals without, with the highest incidence within the first 12 months after tumor diagnosis [38]. However, there is no clear guidance on how to interpret and manage elevated D-dimer levels in cancer patients, leading to decisions often based on empirical experiences and the patient’s clinical context. Considering existing literature, it is important to acknowledge that D-dimer levels in cancer patients may vary according to certain risk factors, such as the location of the primary tumor, the stage of the tumor, and the presence of comorbidities. Additionally, treatment modalities such as chemotherapy, antiangiogenic therapy, surgery, the use of central venous catheters, and hospitalization contribute to the predisposition to thrombosis in these patients [39]. All these factors have the potential to introduce bias into the results and the so-called “optimized” cut-off values. Laboratory biomarkers that can predict the risk of VTE in cancer patients include thrombocytosis or leukocytosis, tissue factor, soluble P-selectin, and D-dimer [40, 41].

In the context of excluding DVT, we came across reports that assessed the cost-effectiveness of combining pretest probability with D-dimer testing and ultrasound. All the studies' findings indicate that using D-dimer as an initial test, followed by ultrasound when necessary, leads to cost savings [16, 42–44]. Even in the context of excluding the diagnosis of PE, diagnostic strategies that incorporate D-dimer testing were found to be cost-effective compared to strategies that do not include D-dimer testing [16].

D-dimer assays used in this context require high sensitivity and, more importantly, an NPV close to 100% to safely exclude VTE when D-dimer levels are below the cut-off. Specificity should also be maximized to minimize false positives. However, since D-dimer levels can be elevated in various clinical situations such as inflammation, myocardial infarction, congestive heart failure, acute aortic dissection, and advanced age, the use of a fixed cut-off for D-dimer in these patient groups is questionable [17, 45]. Various approaches have been explored to address this issue, often involving higher cut-off levels for D-dimer in elderly patients [30]. To improve specificity without significantly reducing sensitivity, an age-adjusted cut-off value was introduced for patients aged 50 or older (age-adjusted cut-off value = age × 10 µg/L) [46–48].

In our retrospective comparative analysis to evaluate potential cost savings by using different D-dimer cut-off values in cancer patients with suspected VTE and a non-high pretest probability, the inverse age-adjusted cut-off method emerged as the most reliable approach for excluding VTE with a PLR of 2.9 at a very low NRL. This method yielded total savings of €5131 and demonstrated the best balance between specificity, sensitivity, and NPV. Moreover, the age-adjusted cut-off method achieved even greater cost savings, totaling €9363. This method also displayed favorable sensitivity and NPV values, although slightly lower than those of the inverse age-adjusted method. De Pooter et al. also demonstrated that the most effective approach was utilizing an age-adjusted cut-off level determined by multiplying the patient's age by 10 for individuals over 50 years old [30]. This strategy proved cost-effective in the validation cohort with a reduction of 6.9% in diagnostic costs for PE and a reduction of 5.1% in DVT, compared to the conventional approach using a D-dimer cut-off value of 0.5 mg/L [30]. In our study, we observed a significant cost reduction of 8.1% for PE and 3.4% for DVT using the age-adjusted method, as well as a reduction of 4.6% for PE and slightly less than 1% for DVT using the inverse age-adjusted method.

Using data from the United States would result in greater savings in our study. With an estimated patient volume of 5475 per year, the inverse age-adjusted method could save $171,453, and the age-adjusted method could save $324,503. In a related study, Blondon et al. assessed the cost-effectiveness of the age-adjusted D-dimer cut-off compared to the standard cut-off in patients with suspected PE and a non-high pretest probability using a decision tree model [49]. With an annual count of 3 million suspected PE cases, the findings demonstrated that adopting an age-adjusted cut-off resulted in a minor reduction in quality-adjusted life-years (QALY) alongside significant cost savings, estimated between $75 million and $98 million per year for the U.S. healthcare system [49].

Research on the usefulness of D-dimer testing to exclude VTE in cancer patients remains limited. Although it's possible that D-dimer levels below the standard rule-out threshold can effectively eliminate VTE in these patients, there exists limited data on the percentage of cancer patients meeting the rule-out criteria. Additional investigations are necessary to verify these observations. This study acknowledges several limitations that should be considered. First, it has been conducted retrospectively at a single center, which may limit the generalizability of the findings to other settings or patient populations. Second, the measurement of D-dimers was based on the discretion of the attending physician, introducing the possibility of selection bias. This means that certain patients may have been more likely to undergo D-dimer testing, potentially influencing the results. Cost-minimization analyses are also reliant on assumptions that have inherent limitations. Furthermore, more data from prospective trials are necessary to evaluate D-dimers as a quantitative biomarker for ruling in VTE, not only in cancer patients but also in other patient populations. Conducting prospective and multi-center studies would offer a more comprehensive evaluation of the economic implications of testing a larger cohort of cancer patients for D-dimer levels upon their admission to the emergency department. Overall, while the study provides valuable insights, it is essential to consider these limitations when interpreting the results and to conduct further research to validate the findings.

## Conclusions

The diagnosis of VTE remains challenging due to its non-specific clinical presentation. Additionally, the low incidence of confirmed cases among patients with suspected VTE makes systematic imaging cost-ineffective and potentially harmful due to radiation exposure [30]. In such scenarios, a systematic application of D-dimer testing would likely enhance the economic balance, albeit at the expense of false positives arising from its relatively low specificity. Our study suggests that using an inverse age-adjusted cut-off level for D-dimer may offer a slightly superior diagnostic approach for VTE compared to the conventional strategy. This approach exhibits remarkable attributes, including a PLR of 2.9 at a very low NLR, and a substantial cost reduction of 4.6% for PE. Recognizing the limitations of current diagnostic methods, additional research is essential to explore and develop novel diagnostic strategies and predictive criteria that can reliably identify cancer patients at an elevated risk of VTE development, ultimately improving patient outcomes and the economic balance.

## Data Availability

The data supporting the findings of this study are accessible from the corresponding author upon reasonable request. They are stored in controlled-access data repositories at the University Hospital Frankfurt am Main.

## Abbreviation

CLSI: Clinical and Laboratory Standards Institute
CUS: Compression ultrasound
CTPA: Computed tomography pulmonary angiography
DVT: Deep vein thrombosis
ECG: Electrocardiogram
IQR: Interquartile range
NLR: Negative likelihood ratio
PE: Pulmonary embolism
PLR: Positive likelihood ratio
PTP: Pretest probability
VTE: Venous thromboembolism
